# African American Adults With Hypertension Managing Long COVID And Accessing Healthcare: A Systematic Review

**DOI:** 10.1093/geroni/igaf122.3526

**Published:** 2025-12-31

**Authors:** Raymond Akorlatse, Rae Walker, Raeann LeBlanc, Michael Lepore

**Affiliations:** University of Massachusetts Amherst, Amherst, Massachusetts, United States; University of Massachusetts Amherst, Amherst, Massachusetts, United States; University of Massachusetts Amherst, Amherst, Massachusetts, United States; University of Massachusetts Amherst, Amherst, Massachusetts, United States

## Abstract

Hypertension (HTN) is a significant risk factor for cardiovascular disease (CVD) and stroke, contributing notably to cardiovascular morbidity and mortality in the United States and globally. The emergence of Long COVID (LC) has expanded the scope of managing hypertension as a cardiovascular and chronic disease in Black and African American adults. This study describes how African American adults exposed to COVID-19 have managed HTN and healthcare access since the pandemic’s onset. We applied the Preferred Reporting Items for Systematic Reviews and Meta-Analyses (PRISMA) 2020 guidelines to synthesize existing knowledge. Research articles from 2020-2025 were identified across the Web of Science (Core Collection), MEDLINE (Ovid), and CINAHL databases. English language peer-reviewed studies that reported Social determinants of health (SDOH) factors, COVID-19, HTN, CVD, and healthcare access among Black and African American adults were included. Twenty-three articles were selected. Results revealed common barriers to healthcare, including lack of insurance, transportation challenges, and poor continuity of care. Existing research identified SDOH factors such as employment, redlining, housing insecurity, Medicaid/uninsured, low-income status, low education, rent moratorium, and ethnicity as drivers of health inequities. Though telehealth access and use increased significantly during the pandemic, Black and African American adults continued to face health care access barriers such as lack of access to high-speed internet or broadband, variable levels of digital literacy, and mistrust. Diagnosis of long COVID is a predictor for further health inequities among Black and African American persons managing a CVD diagnosis, geographical location, language, and sociodemographic characteristics (age, sex, gender).

